# Inflammatory cytokine and humoral responses to *Plasmodium falciparum* glycosylphosphatidylinositols correlates with malaria immunity and pathogenesis

**DOI:** 10.1002/iid3.89

**Published:** 2015-11-06

**Authors:** Babacar Mbengue, Birahim Niang, Maguette Sylla Niang, Marie Louise Varela, Becaye Fall, Mouhamadou Mansour Fall, Rokhaya Ndiaye Diallo, Bacary Diatta, D. Channe Gowda, Alioune Dieye, Ronald Perraut

**Affiliations:** ^1^ Service d'Immunologie Université Cheikh Anta Diop de DakarUCADDakarSenegal; ^2^ Unité d'ImmunogénétiqueInstitut Pasteur de Dakar, IPDDakarSenegal; ^3^ Service de RéanimationHôpital Principal de Dakar, HPDDakarSenegal; ^4^ Unité d'ImmunologieInstitut Pasteur de Dakar, IPDDakarSenegal; ^5^ Fédération des laboratoiresHôpital Principal de Dakar, HPDDakarSenegal; ^6^ Department of Biochemistry and Molecular BiologyPennsylvania State University College of Medicine, Milton S. Hershey Medical Center PennsylvaniaHersheyUSA

**Keywords:** cytokines, IgG, parasitemia, *Plasmodium falciparum* GPIs, severe malaria

## Abstract

Pro‐inflammatory cytokines induced by glycosylphosphatidylinositols (GPIs) of *Plasmodium falciparum* contribute to malaria pathogenesis and hence, the naturally acquired anti‐GPI antibody thought to provide protection against severe malaria (SM) by neutralizing the stimulatory activity of GPIs. In previous studies, the anti‐GPI antibody levels increased with age in parallel with the development of acquired immunity, and high levels of anti‐GPI antibodies were associated with mild malaria (MM) cases. In the present study, the relationship between the levels of pro‐inflammatory cytokines and anti‐GPI IgG antibody responses, parasitemia, and the clinical outcomes were evaluated in SM and mild malaria (MM) patients. Sera from a total of 110 SM and 72 MM cases after excluding of ineligible patients were analyzed for the levels of anti‐GPI antibodies, IgG subclasses, and cytokine responses by ELISA. While the total anti‐GPI antibody levels were similar in overall SM and MM groups, they were significantly higher in surviving SM patients than in fatal SM cases. In the case of cytokines, the TNF‐α and IL‐6 levels were significantly higher in SM compared to MM, whereas the IL‐10 levels were similar in both groups. The data presented here demonstrate that high levels of the circulatory pro‐inflammatory, TNF‐α, and IL‐6, are indicators of malaria severity, whereas anti‐inflammatory cytokine IL‐10 level does not differentiate SM and MM cases. Further, among SM patients, relatively low levels of anti‐GPI antibodies are indicators of fatal outcomes compared to survivors, suggesting that anti‐GPI antibodies provide some level of protection against SM fatality.

## Introduction

*Plasmodium falciparum* malaria is one of the major public health problems in tropical regions of the world with ∼250 million clinical cases and ∼600,000 deaths annually. Of the total global malaria morbidity and mortality, >80% of clinical cases and ∼90% of deaths occur in sub‐Saharan Africa, particularly in children under five years. The widespread deployment of long‐lasting insecticide treated bed nets, the availability of effective artimisinin‐based anti‐malarial combination therapy and the improved disease prevention efforts have dramatically decreased the malaria burden in many parts of Africa 1. However, a consequence of this prevention strategy is the development of drug‐resistant parasites and mosquito vectors 2, 3, 4. As immunity to malaria wanes away quickly, another consequence of decrease in immune protection is due to reduced exposure to the infection and consequent lack of periodic immune boosting. Thus, malaria burden is expected to be on the raise unless newer prevention and treatment strategies are developed. One approach toward this goal would be to gain mechanistic insight into the processes involved in malaria pathogenesis and immune responses.

Although failure of the immune system to control rapid parasite replication and consequent excessive inflammatory responses is considered as contributing factors to malaria immunopathology 5, 6, 7, the underlying mechanisms are not fully understood. In fact, the ability of *P. falciparum* infected red blood cells to sequester in the deep endothelia of vital organs, including brain, liver, and spleen, leads to the accumulation of high concentrations of toxic parasite components at sites of sequestration, resulting in strong induction of pro‐inflammatory cytokine production, endothelial damage, organ dysfunction, and life threatening pathological conditions. Several studies have shown that glycosylphosphatidylinositols (GPIs) of parasites is one of the parasite toxic factors that contribute to malaria pathogenesis 8, 9, 10, 11, 12, 13. This idea was based on the ability of GPIs to induce production of TNF‐α, IL‐1, IL‐6, and IFN‐γ in macrophages and cause symptoms reminiscent of severe malaria (SM) illnesses, including pyrexia, hypoglycemia, and lethal cachexia in animals 12. The idea was further substantiated by the fact that immunization with parasite GPIs reduced the inflammation associated with acute *Plasmodium berghei* infection in mice 13, and that GPIs activate CD36‐, TLR2‐, and TLR4‐dependent signaling cascades to induce inflammatory cytokines and nitric oxide production from human macrophages in vitro 8, 10, 11. However, the relationship between GPI‐induced inflammatory cytokines, such as TNF‐α, IL‐6 and IL‐1, and IgG responses and outcome in SM in human is not well understood. We have previously reported that in urban hypoendemic area, low anti‐GPI IgG responses correlate with cerebral malaria (CM) cases compared to relatively high levels of anti‐GPI antibodies in patients with mild malaria (MM) 14. The aim of the present study was to examine, in SM patients during three days of hospitalization compared to MM cases, the relationship between the magnitude of peripheral inflammatory cytokine responses, the levels of anti‐GPI IgG antibody responses, the parasitological characteristics, and the clinical outcome.

## Materials and Methods

### Study area and epidemiologic context

The study was performed in Dakar, a malaria hypoendemic region, having a low level of seasonal transmission with an average of 0.5–1 infecting bite/person/year during the rainy season, September–December 15, 16. The main malaria vector described is *Anopheles arabiensis*, and *P. falciparum* is the most widespread malaria species (98% of cases) 15, 17. Malaria transmission is unstable and characterized by a highly variable density of vector and an entomological infection rate (EIR) of 3–9 infective bite per individual per year during the rainy season in some locations 17. Previous studies in this area have revealed that malaria affects all age groups with the highest prevalence occurring in children 14, 15. A mean incidence of 2.4% of clinical accesses has been observed with no difference between adults and children 15.

### Study design, ethic statements, and procedures

Patient positive for *P. falciparum* parasites on blood film or by rapid diagnostic test were enrolled after written and verbal informed consents were obtained from the patients or their relatives. Ethical approvals were obtained from the investigators' institutions and the National Ethical Committee from Ministry of Health.

The presence of *P. falciparum* parasitemia was determined by microscopic examination of thin and thick smears prior to anti‐malarial treatment. A questionnaire with clinical history and demographic information was recorded. On the first day of hospital attendance/admission, complete clinical parameters including parasite density, hematologic, and clinical characteristics were determined by the hospital's clinical laboratory. Blood samples used in this study were those collected after parasitologic and clinic profiles were determined. Samples were centrifuged, plasma aliquoted, and stored at −80°C until testing. The following criteria were used in stratifying patients into two different groups.

Life‐threatening SM was characterized by at least one of ten WHO criteria including cerebral symptoms, coma, severe anemia, respiratory distress, liver dysfunction, renal impairment, lactic acidosis, hypoglycemia, renal, and non‐cardiogenic pulmonary oedema 18. MM cases were defined as those with positive blood smear for *P. falciparum* and having fever, and no symptoms of SM.

The study was performed at the Hospital Principal of Dakar during September to December in two successive years 2003 and 2004. *P. falciparum*‐infected SM patients were admitted to the Intensive Care Unit of the hospital. All patients were managed by the same medical staff and according to the hospital treatment protocol based on the national recommendations, intramuscular quinine 20 mg/kg followed by 20 mg/kg every 8 h. Patients were examined every 4 h for the first 24 h and every 6 h thereafter. Patients with fever and clinical signs of cerebral impairment underwent a lumbar puncture to rule out meningitis, and patients with mixed infection of malaria parasites were excluded.

**Figure 1:**
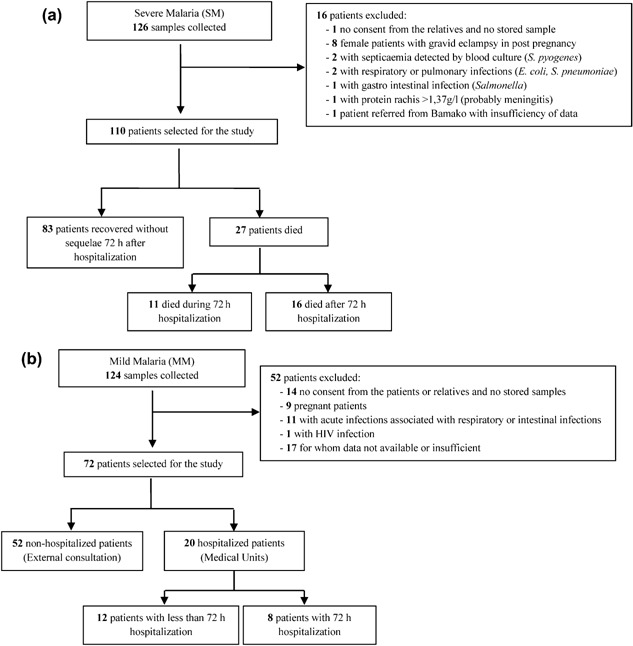
Flow chart of the recruitment for patients with severe (A) or mild malaria (B). A summary of the recruitment and the reasons of exclusion of patients (16 SM and 52 MM patients) are outlined.

As shown in Figure 1, of a total of 126 SM patients initially enrolled, 16 patients who had other infections prior to the onset of SM episode were excluded; thus, 110 patients were included in this study. Of these 110 SM patients, 83 were recovered with no sequel, 27 had fatal outcomes, and 11 died prior to the third assessment day. The causes of death were due to SM (Fig. 1A).

A total of 124 MM patients who are treated at the outpatient clinic of the hospital were initially enrolled. Of these, 52 were excluded based on the criteria outlined in Figure 1B, and the remaining 72 were included in the study. Blood samples from MM patients were obtained on the day of clinical presentation.

### *P. falciparum* GPIs

*P. falciparum* GPIs were isolated from the late trophozoite and schizont stages of cultured *P. falciparum* (FCR3 strain), and purified by HPLC as described earlier 19, 20.

### Anti‐GPI IgG ELISA

ELISA for assessing anti‐GPI IgG levels was performed as reported previously 14, 20. The 96‐wee flat‐bottomed Immulon‐4 ELISA plates (Dynatech, Spingfield, VA) were coated with parasite GPIs (2 ng GPIs) and blocked with phosphate‐buffered saline (PBS) containing 5% bovine serum albumin (BSA). Plates were incubated with 50 μL of plasma samples were diluted 1:100 in PBS having 1% bovine serum albumin. Pooled plasma from European volunteers with no exposure to malaria and naive Senegalese plasma was used as a negative control and pooled plasma from immune individuals living in the holoendemic village of Dielmo was used as a positive control 22. Detection of the bound anti‐GPI antibodies was with peroxidase‐labeled goat anti‐mouse IgG (Cappel Laboratories Inc., Cochranville, PA, USA; diluted 1:2000) as described previously 14. Samples were analyzed in duplicate. Responses were expressed as OD ratios: OD_serum sample_/OD_negative control_. Serum samples with OD ratios greater than two (which was over the OD of naive control + 2 SD) were considered as seropositive. For positive controls, OD was ∼0.5, giving an OD ratio of 10.

IgG subclass analysis by ELISA was performed as described previously using human sub‐class specific mouse mAbs 20, 21, 23. The detection was by using peroxidase‐labeled goat anti‐mouse IgG (1:2000) as outlined above.

### Determination of Serum Cytokine levels

TNF‐α, IL‐6, and IL‐10 were analyzed by using ELISA kit according to the manufacturer's instructions (Pharmingen^®^, San Diego, CA). Flat‐bottomed 96‐well plates (Maxisorp®, Nalge Nunc International Rochester, NY, USA) were coated with capture antibody diluted at 1:250 in PBS for all the cytokines. The wells were blocked with a solution of 1% of BSA and incubated with 1:2‐diluted plasma samples, and positive and negative controls. After 1 h, wells were incubated biotinylated antibody (1:250 dilution for anti‐TNF and anti‐IL‐6 antibodies, and 1:500 dilution for anti‐IL‐10 antibody) followed by alkaline phosphate‐conjugated avidin in Tris buffer, pH 7, containing 1% BSA. The detection was with o‐toluidine substrate (Sigma Chemical cie, St. Louis, MO, USA) in the presence of hydrogen peroxide. Color development was stopped with 4 N sulphuric acid (Prolabo, Fontanay sous Bois, France). The optical density was measured at 450 nm in a plate reader (Tecan^®^, GmbH, Salzburg, Austria). Analysis was performed in duplicate. Cytokines concentrations (pg/mL) were determined by using the standard curve and multiplied by the dilution factor. The detection limit of each of cytokine assays was 7.8 pg/mL for IL‐10 and TNF‐α, and 4.7 pg/mL for IL‐6; values below this level were considered zero.

### Statistical analysis

The antibody responses were not normally distributed (Shapiro test) and hence, non parametric tests were used for analyses. Continuous variables were compared using Mann–Withney, Spearman's rho, and Kruskal–Wallis tests. Inter‐assay comparisons of results were evaluated using Wilcoxon rank test. The Fisher's exact test and the chi‐square were used to compare prevalence of responses between groups. The result was considered significant at the 5% level and the Bonferonni correction was made for multiple comparisons. Analyses were performed by using Statview 5.0® (Abacus Concept Inc., San Francisco, CA, USA) and Sigmaplot 12.0® (Systat Software Inc., St. Jose, CA, USA).

## Results

### Profiles of IgG and IgG subclass responses against *P. falciparum* GPIs

Plasma from both SM and MM groups were tested for total anti‐GPI IgG and IgG subclasses (IgG_1–4_). Total anti‐GPI IgG responses are shown in Table 1. On the day of admission, there were no significant differences in the seroprevalence and levels of antibodies between SM and MM groups. However, anti‐GPI IgG levels were significantly different in the SM group of patients stratified according to the clinical outcome, surviving (Surv. SM) versus fatal cases (Fatal SM).

**Table 1:** Sero‐prevalence and levels of IgG antibodies responses against GPI.

Patients	No.	Age	IgG to GPI day 0			IgG to GPI day 1				IgG to GPI day 2			
		Mean [range]	%^2^	Mean [range]	*P* 3 ^,^ 4	No	%^3^	Mean [range]	*P*	No	%^2^	Mean [range]	*P* 3 ^,^ 4
Surviving SM^1^	83	28 [8–73]	63	3.7 [1–12.7]		82	52	4.1 [1–27.5]		77	50	3.6 [1–26.5]	
Fatal SM^1^	27	33 [11–74]	41	2.6 [1–11.5]	0.02	22	27	2.2 [1–9.8]	0.01	16	44	3.4 [1–20.0]	NS
All SM	110	29 [8–74]	57	3.4 [1–12.7]		104	47	3.7 [1–27.5]		93	49	2.5 [1–26.5]	
Mild malaria	72	30 [2–77]	49	3.8 [1–21.6]	NS	Not available				Not available			

1 Patients with severe cerebral malaria (SM); No., Number of patients.

2 Prevalence of responders to GPI that is individuals with IgG responses >2 OD ratio.

3 Statistical comparison between surviving versus fatal SM and all SM versus MM.

4 Comparison of prevalence between surviving versus fatal SM and all SM versus MM by Fisher's exact test was not significant.

**Figure 2:**
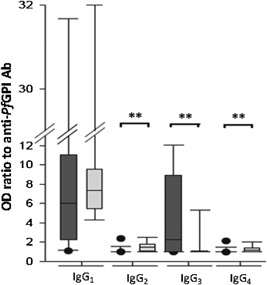
IgG subclass responses to GPIs. Isotype‐specific IgG responses against GPIs in a group of 40 patients are shown as box‐whisker plots, representing median with 25th and 75th percentile (boxes), and 10th and 90th percentiles (whiskers) for a subset of patients with SM (black bars) and MM (gray bars). Brackets with asterisk indicate significant different levels of IgG subclass responses (**P < 0.01). Black bars, 23 SM cases; gray bars, 17 MM patients.

IgG subclass antibody responses to GPIs were determined on the day of admission for a subset of 40 patients, of which 23 were SM patients 11–65 years old (mean age 26.8 years), and 17 were MM patients 2–73 years old (mean age 27.6 years). As shown in Figure 2, in this subgroup, anti‐GPI IgG responses were predominantly IgG_1_ and IgG_3_. No significant differences in IgG_1_ levels were found between SM and MM groups. However, IgG_3_ antibody levels were significantly higher in SM (mean OD ratio 4.6; range 1.0–15.8) than in MM (mean OD ratio 1.7; range 1.0–8.9) (*P* < 0.001) individuals. The levels of IgG_2_ and IgG_4_ were generally low in both groups but significantly higher in MM patients compared with SM patients (*P* < 0.001) (Fig. 2). No correlation was found between IgG subclasses antibody responses to GPI and ages of individuals in MM and SM subgroups.

### Relationship between IgG responses against GPIs and parasitemia

Global analysis of association between anti‐GPI IgG levels and parasitemia measured on the day of admission (day 0), showed statistically no significant association between the two parameters in SM and MM patients. Further, similar results were observed when analyses were done according to the age of patients, in each clinical group (SM and MM). Only individuals harboring high level of parasitemia (>5%) exhibited high levels of anti‐GPI antibodies on days 0, 1, and 2.

### Plasma cytokines levels in different study groups: correlation with disease severity and clinical outcome

To evaluate the prognostic significance of pro‐inflammatory and anti‐inflammatory cytokines in *P. falciparum* malaria, levels of TNF‐α, IL‐6, and IL‐10 in the blood plasma of SM and MM groups were analyzed.

The levels of pro‐inflammatory cytokines, TNF‐α and IL‐6 were higher in SM group than MM cases (*P* < 0.05); approximately threefold for TNF‐α and twofold for IL‐6 (Table 2). There was no statistically significant difference for IL‐10 levels between MM and SM patients (*P* = 0.106).

**Table 2:** Levels and kinetic profiles of cytokines in SM.

		Cytokine level day 0			Cytokine level day 1			Cytokine level day 2		
Cytokine	Patients	*N*	Mean [range]	*P* 2	*N*	Mean [range]	*P*	*N*	Mean [range]	*P*
TNF‐α	Surv. SM^1^	83	122 [2–2492]		79	81 [1–2569]		72	51 [1–2244]	
	Fatal SM^1^	26	147 [2–2114]	0.02	22	15 [1–109]	<0.01	16	27 [1–193]	NS
	All SM	109	128 [2–2492]		101	67 [1–2569]		88	47 [1–2244]	
IL6	Surv. SM^1^	82	155 [1–2315]		79	169 [1–1565]		72	94 [0–473]	
	Fatal SM^1^	26	609 [3–5925]	0.02	22	1526 [4–8062]	<0.01	16	709 [22–6167]	NS
	All SM	108	264 [1–5925]		101	465 [1–8062]		88	206 [0–6167]	
IL10	Surv. SM^1^	82	463 [7–3502]		79	367 [5–2192]		72	378 [8–3467]	
	Fatal SM^1^	26	745 [12–3624]	0.02	22	719 [7–2746]	<0.01	16	520 [25–2015]	NS
	All SM	109	531 [7–3624]		101	377 [5–2746]		88	404 [8–3467]	

1 Patients with severe malaria (SM): Surv., surviving patients; *N*, number of determinations.

2 Statistical comparison between surviving versus fatal SM.

**Figure 3:**
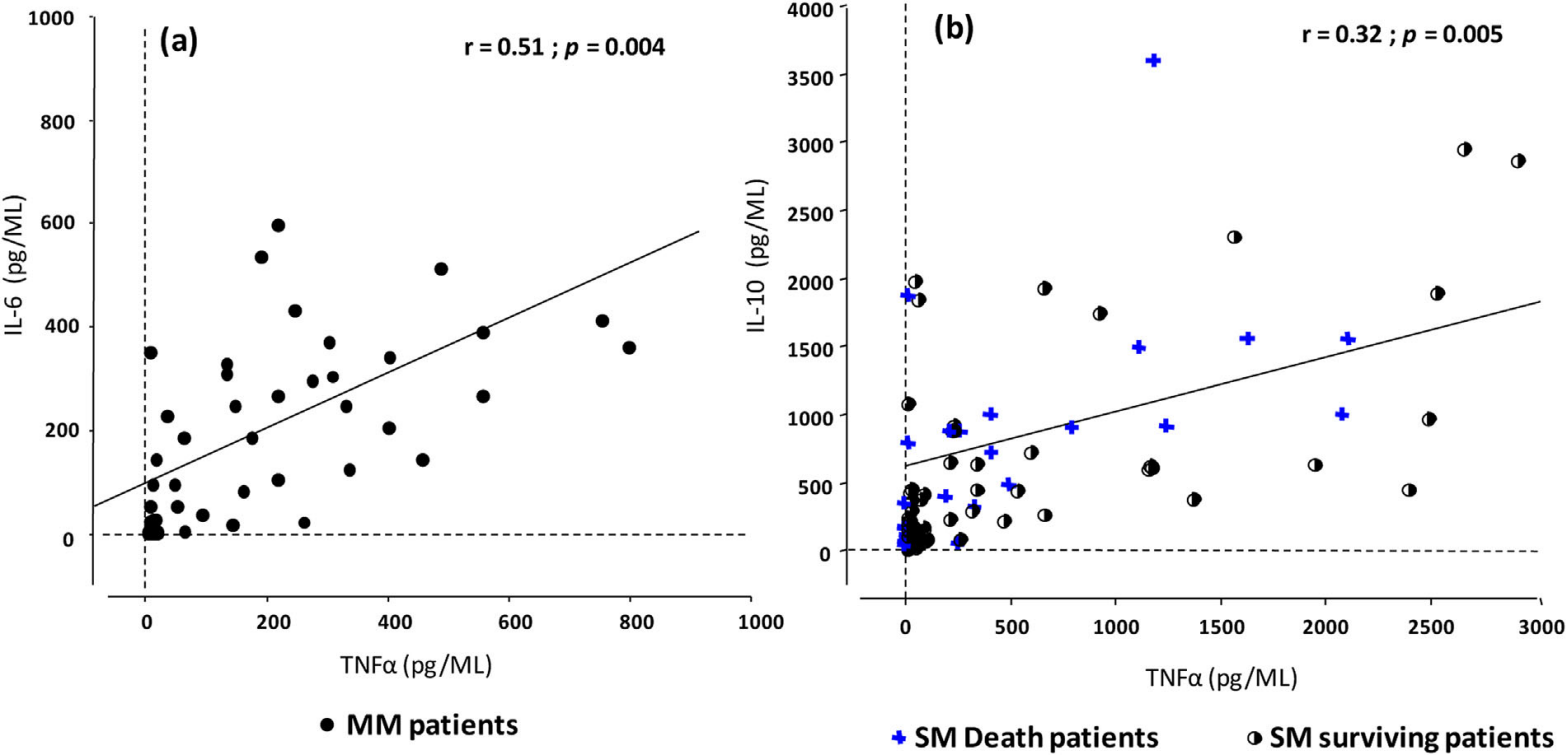
Correlations between pro‐inflammatory and anti‐inflammatory cytokines in MM (a) and SM (b) groups. The positive correlation between cytokines plasma levels is shown as a bivariate dot plot: IL‐6 and TNF‐α in MM group (a), and IL‐10 and TNF‐α in SM group (b). Statistical analyses were done by non parametric Spearman Rank Test. Only the MM patients are shown in Figure 3a. Figure 3b shows surviving SM patients (circles) and fatal outcome (blue cross).

To evaluate the relationship between pro‐inflammatory and anti‐inflammatory plasma cytokine levels, we analyzed the correlation among these markers according to disease severity and progression of the disease. In MM group, IL‐6 and TNF‐α levels were positively correlated (*r* = 0.51; *P* = 0.004) (Fig. 3a). Conversely, a positive correlation was found between TNF‐α and IL‐10 plasma concentrations in SM patients on day 0 (*r* = 0.32, *P* = 0.005). Furthermore, the correlation between TNF‐α and IL‐10 levels was higher in patients who died (*r* = 0.45, *P* = 0.010) than patients who recovered (*r *= 0.21, *P* = 0.031) and these correlations disappeared two days after admission (Fig. 3b).

According to previous studies, the ratio of anti‐inflammatory (IL‐10) to inflammatory (TNF‐α) is a potentially useful parameter indicator in cerebral malaria. Low IL‐10:TNF‐α plasma level ratios were found to be a risk factor for both cerebral malaria and severe anemia 24, 25. Here, the ratio of anti‐inflammatory cytokine IL‐10 to pro‐inflammatory cytokine TNF‐α was significantly higher in MM than SM group (41.3 ± 9.4 vs. 29.2 ± 6.7; *P* = 0.011) on the admission day, in agreement with the clinical outcome of SM patients. The IL‐10: TNF‐α ratio did not significantly varied at day 2.

No relationship was found between parasitemia, and either TNF‐α, IL‐6 and IL‐10 plasma levels or IL‐10:TNF‐α ratio in the different categories of patients.

### Relationship between plasma cytokine levels and anti‐GPI IgG responses

The correlation between anti‐GPI IgG antibody levels and cytokine in combination with clinical and parasitological parameters was analyzed.

**Figure 4:**
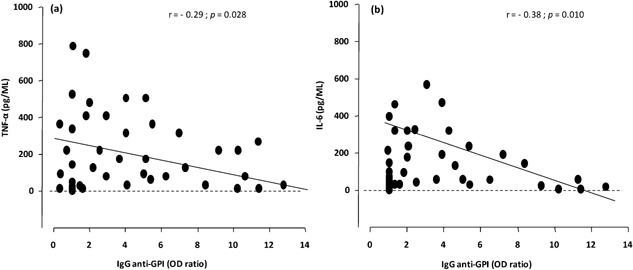
Relationship between anti‐GPI IgG responses and plasma cytokines, TNF‐α (a) IL‐6 (b), in MM group. The correlation between cytokines plasma levels (pg/ML) and IgG anti‐GPI levels (OD ratio) is plotted in MM patients, that is, anti‐GPI IgG responses and TNF‐α (a) and anti‐GPI IgG and IL‐6 (b). Results from statistical analyses done by non parametric Spearman rank test are indicated.

In MM group, IgG anti‐GPI levels were inversely correlated to TNF‐α (*r* = −0.29; *P* = 0.028) (Fig. 4a) and IL‐6 (*r* = −0.38; *P* = 0.010) levels (Fig. 4b). Among the IgG subclasses, only IgG_1_ levels showed a significant inverse correlation with TNF‐α (*r *= −0.37; *P* = 0.019) and IL‐6 (*r* = −0.41; *P* = 0.021) levels in MM group.

**Figure 5:**
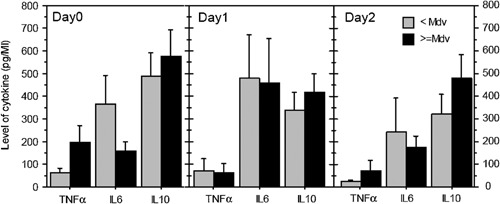
Profile of cytokines levels as function of anti‐GPI IgG responses in SM groups. Mean values ± SE of cytokines levels in pg/mL are shown as histograms. The black bars represent SM patients with IgG anti‐GPI OD ratio ≥ median value (Mdv) and the grey bars represent SM patients with IgG anti‐GPI OD ratio ˂ median value. Results for TNF‐α, IL‐6, and IL‐10 are shown on days 0, 1, and 2.

In SM group, individual cytokine levels are plotted in Figure 5 as function of dichotomized anti‐GPI antibody levels under and above the median value of OD ratio. On the day of admission, there was a trend toward higher concentration of TNF‐α and lower concentration of IL‐6 in SM patients with high levels of anti‐GPI IgG, but the values were not statistically significant. No difference was globally detected for IL‐10 concentrations. This profile was visible on day 3 with significant lower levels of cytokines, but not on day 2.

## Discussion

Malaria infection triggers innate and adaptive immune responses. Although complete details of the host‐pathogen interactions that lead to immune responses are lacking, a number of studies have shown that immune response contribute to both disease pathogenesis and protective immunity 5, 6. One of the questions in understanding anti‐disease immunity in human malaria is the contribution of anti‐GPI antibodies compared to antibodies against other parasite factors involved in protection against malaria illnesses. People living in endemic areas have anti‐GPI antibodies 8, 20, 24, which might provide some degree of protection against malaria symptoms. High levels of the anti‐GPI antibodies have been correlated with resistance to clinical symptoms, such as anemia and fever 19, and hence these antibodies were suggested to be an important factor in the protective immunity to malaria 8, 11. Consistent with these results, lower levels of anti‐GPI were observed among Senegalese adults with cerebral malaria compared to individuals with uncomplicated malaria 14. However, the results presented here indicate the absence of significant differences either in prevalence or the levels of anti‐GPI IgGs between SM and MM groups. These results agree with those of previous studies reporting no significant differences in anti‐GPI antibody responses between Gambian children having SM or MM 25 and higher levels of anti‐GPI IgG antibodies were found in Malian children with CM 24. A possible explanation for these contrasting results is that the recruitment of severe urban malaria is highly heterogenic regarding the initial onset of the infection and the delay before hospitalization in the severe cases. We had previously reported that delay of admission appeared as significantly increased SM 14, 26.

Our observation that anti‐GPI IgG responses do not correlate with parasitemia either in MM group or SM group agrees with the results of a previous study reporting a lack of association between anti‐GPI antibodies and the ability of Papua New Guinean children to tolerate high‐density parasitemia 8. Interestingly, we observed a substantial increase of IgG levels after the beginning of the treatment in SM patients with an elevated initial parasitemia on day 0. This is in line with the profile of fluctuation of anti‐GPI levels at the time of the measure in SM that relates to the higher parasite load.

Some previous studies had reported associations between levels of IgG or IgG subclass Ab against GPI and age of subjects living in hyper endemic areas. In our study, these associations were not observed. Discordances can be explained firstly by the low endemicity in urban area of Dakar 17 and the second explanation can be the fact that our study population was constituted by symptomatic malaria patients and as described previously no significant difference of IgG anti‐*P. falciparum* levels were reported between adults and children hospitalized patients 26, 27.

An important observation of this study is the difference of anti‐GPI total IgG levels observed between surviving and fatal SM. Surviving individuals had significant high levels of IgG to GPI than fatal cases on day 0 and day 1, but not on day 2. This suggests a possible protection‐associated effect of these antibodies against fatal outcome. In Thailand, Brasseur et al. found that antibody titers significantly differed between patients who died of SM and those who recovered despite both groups were adequately treated 28. Furthermore, the IgG subclass antibody responses found in this study are in agreement with the results of previous studies, reporting that short‐lived anti‐GPI IgG_1_ and IgG_3_ responses predominate in asymptomatic individuals with circulating parasites 8.

A number of cytokines and bioactive proteins, such as procalcitonin were previously found to contribute to adverse prognosis in human SM 7, 29, 30. In agreement, we found elevated levels of pro inflammatory cytokines in patients with SM; approximately two‐ and threefolds higher for IL‐6 and TNF‐α compared to MM cases at the day of admission, but no significant difference of IL‐10 plasmatic levels between the two groups of patients. These results suggest that TNF‐α and IL‐6 are associated with the pathogenesis of human SM. These results are in line with several other observations, reporting high circulating levels of pro‐inflammatory cytokines, such as TNF‐α 7, 29, 31 and IL‐6 6, 7, 29, 32, 33 during the acute phase of SM in human, as well as in experimental models 31.

GPI has been proposed as a putative malaria toxin eliciting the inflammatory response contributing to pathogenesis 12. On one hand, GPIs initiate the release of pro‐inflammatory cytokines including TNF‐α, IL‐1, and IL‐6, on the other hand antibodies against GPI mediate an inhibition of cytokines release as demonstrated in vitro 34. However, no correlation was detected in the present work between anti‐GPI IgG levels and plasmatic concentrations of pro‐inflammatory cytokines, TNF‐α, and IL‐6, during the three days survey. Several reasons could explain such result. First, the limitation of ex‐vivo cytokines measurements as underlined in several clinical studies 35. Most cytokines are cleared very rapidly (half‐lives of minutes), so that circulating levels are influenced by the timing of sampling and the duration the stimulus for synthesis 29. The half‐life may be prolonged by binding to soluble receptors and also by decreased renal or hepatic clearance.

Another explanation of the non significant relationship between anti*‐*GPI IgG levels and cytokines concentrations may imply the signaling pathway of GPI effect that can be stimulated by others toxins from the parasite. Several studies demonstrated that malaria infection induces activation of Toll‐like receptor (TLR1, TLR2 TLR4, and TLR9) and involvement of anti‐TLR2 and anti‐TLR4 antibodies 36, 37, 38. Krishnegowda et al. have confirmed that GPIs are recognized mainly by TLR2 and to a lesser extent by TLR4 10. Recently, TLR9 and MyD88 were shown implicated in the regulation of anti‐*P. falciparum* immune response via dendritic cells after activation by the parasite DNA 11, 39. In both SM and sepsis, a variety of toxins triggers the activation of MAPK and NFκB signalling pathways to induce the release of host immune factors that include cytokines, such as TNF, IL‐6, IL‐1, oxygen free radical. Therefore, GPI is only one of the multiple malaria toxins that stimulate this signalling pathway. A number of pro‐inflammatory components of *P. falciparum* have been described that include hemozoin 40, parasite membrane‐derived microparticles 41, 42, RESA 43, protein DNA complexes 44, and uric acid 45.

## Conclusion

Overall, the results of this study reveal that higher levels circulatory pro‐inflammatory cytokines, such as TNF‐α and IL‐6, but not the levels of anti‐inflammatory cytokines, such as IL‐10 are indicators of SM. Additionally, although the total anti‐GPI antibody levels were similar in SM and MM patients, interestingly there was a clear correlation between surviving SM cases compared to SM cases with fatal outcomes; that is, the anti‐GPI antibody levels were significantly higher in the surviving SM group than SM group who died. The low levels anti‐GPI IgG in fatal cases raise the question whether this is due to insufficient in vivo IgG production or excessive consumption of antibodies during the course of disease severity. Further studies are required to elucidate the mechanisms that underlie the dynamic of anti GPI antibody responses and their relation to pro‐inflammatory cascade effects.

## Authors Contribution

RP, DCG, and AD designed the study. BM, BN, BF, MF, and BD were in charge of recruitment of patients. BM and RP performed the tests and were in charge of database management and the plasma database. DCG provided the GPIs. BM and RP conducted data analyses with input from RN, MSN, MLV, and AD. BM and RP drafted the manuscript with input from AD and DCG. All authors read and approved the final manuscript.

## Conflict of Interest

We know of no competing interests that exist.

## References

[1] WHO . World Malaria Report 2013. 2014; Geneva 1–178.

[2] Dondorp, A. M. , F. Nosten , P. Yi , D. Das , A. P. Phyo , J. Tarning , K. M. Lwin , F. Ariey , W. Hanpithakpong , S. J. Lee , et al. 2009 Artemisinin resistance in *Plasmodium falciparum* malaria. N. Engl. J. Med. 361:455–467. 19641202 10.1056/NEJMoa0808859 PMC3495232

[3] Noranate, N. , R. Durand , A. Tall , L. Marrama , A. Spiegel , C. Sokhna , B. Pradines , S. Cojean , M. Guillotte , E. Bischoff , et al. 2007 Rapid dissemination of *Plasmodium falciparum* drug resistance despite strictly controlled antimalarial use. PLoS ONE 2:e139. 17206274 10.1371/journal.pone.0000139 PMC1764034

[4] Tun, K. M. , M. Imwong , K. M. Lwin , A. A. Win , T. M. Hlaing , T. Hlaing , K. Lin , M. P. Kyaw , K. Plewes , M. A. Faiz , et al. 2015 Spread of artemisinin‐resistant *Plasmodium falciparum* in Myanmar: a cross‐sectional survey of the K13 molecular marker. Lancet Infect. Dis. 15:415–421. 25704894 10.1016/S1473-3099(15)70032-0 PMC4374103

[5] Clark, I. A. , A. C. Budd , L. M. Alleva , and W. B. Cowden . 2006 Human malarial disease: a consequence of inflammatory cytokine release. Malar. J. 5:85. 17029647 10.1186/1475-2875-5-85 PMC1629020

[6] Clark, I. A. , and K. A. Rockett . 1994 The cytokine theory of human cerebral malaria. Parasitol. Today 10:410–412. 15275552 10.1016/0169-4758(94)90237-2

[7] Day, N. P. , T. T. Hien , T. Schollaardt , P. P. Loc , L. V. Chuong , T. T. Chau , N. T. Mai , N. H. Phu , D. X. Sinh , N. J. White , et al. 1999 The prognostic and pathophysiologic role of pro‐ and antiinflammatory cytokines in severe malaria. J. Infect. Dis. 180:1288–1297. 10479160 10.1086/315016

[8] Boutlis, C. S. , D. C. Gowda , R. S. Naik , G. P. Maguire , C. S. Mgone , M. J. Bockarie , M. Lagog , E. Ibam , K. Lorry , and N. M. Anstey . 2002 Antibodies to *Plasmodium falciparum* glycosylphosphatidylinositols: inverse association with tolerance of parasitemia in Papua New Guinean children and adults. Infect. Immun. 70:5052–5057. 12183552 10.1128/IAI.70.9.5052-5057.2002 PMC128285

[9] Brattig, N. W. , K. Kowalsky , X. Liu , G. D. Burchard , F. Kamena , and P. H. Seeberger . 2008 *Plasmodium falciparum* glycosylphosphatidylinositol toxin interacts with the membrane of non‐parasitized red blood cells: a putative mechanism contributing to malaria anemia. Microbes Infect. 10:885–891. 18657459 10.1016/j.micinf.2008.05.002

[10] Krishnegowda, G. , A. M. Hajjar , J. Zhu , E. J. Douglass , S. Uematsu , S. Akira , A. S. Woods , and D. C. Gowda . 2005 Induction of proinflammatory responses in macrophages by the glycosylphosphatidylinositols of *Plasmodium falciparum*: cell signaling receptors, glycosylphosphatidylinositol (GPI) structural requirement, and regulation of GPI activity. J. Biol. Chem. 280:8606–8616. 15623512 10.1074/jbc.M413541200 PMC4984258

[11] Patel, S. N. , Z. Lu , K. Ayi , L. Serghides , D. C. Gowda , and K. C. Kain . 2007 Disruption of CD36 impairs cytokine response to *Plasmodium falciparum* glycosylphosphatidylinositol and confers susceptibility to severe and fatal malaria *in vivo* . J. Immunol. 178:3954–3961. 17339496 10.4049/jimmunol.178.6.3954

[12] Schofield, L. , and F. Hackett . 1993 Signal transduction in host cells by a glycosylphosphatidylinositol toxin of malaria parasites. J. Exp. Med. 177:145–153. 8418196 10.1084/jem.177.1.145 PMC2190877

[13] Schofield, L. , M. C. Hewitt , K. Evans , M. A. Siomos , and P. H. Seeberger . 2002 Synthetic GPI as a candidate anti‐toxic vaccine in a model of malaria. Nature 418:785–789. 12181569 10.1038/nature00937

[14] Perraut, R. , B. Diatta , L. Marrama , O. Garraud , R. Jambou , S. Longacre , G. Krishnegowda , A. Dieye , and D. C. Gowda . 2005 Differential antibody responses to *Plasmodium falciparum* glycosylphosphatidylinositol anchors in patients with cerebral and mild malaria. Microbes Infect. 7:682–687. 15848275 10.1016/j.micinf.2005.01.002

[15] Pages, F. , G. Texier , B. Pradines , L. Gadiaga , V. Machault , F. Jarjaval , K. Penhoat , F. Berger , J. F. Trape , C. Rogier , et al. 2008 Malaria transmission in Dakar: a two‐year survey. Malar. J. 7:178. 18796138 10.1186/1475-2875-7-178 PMC2556698

[16] Trape, J. F. , E. Lefebvre‐Zante , F. Legros , G. Ndiaye , H. Bouganali , P. Druilhe , and G. Salem . 1992 Vector density gradients and the epidemiology of urban malaria in Dakar, Senegal. Am. J. Trop. Med. Hyg. 47:181–189. 1354414 10.4269/ajtmh.1992.47.181

[17] Gadiaga, L. , V. Machault , F. Pages , A. Gaye , F. Jarjaval , L. Godefroy , B. Cisse , J. P. Lacaux , C. Sokhna , J. F. Trape , et al. 2011 Conditions of malaria transmission in Dakar from 2007 to 2010. Malar. J. 10:312. 22018223 10.1186/1475-2875-10-312 PMC3216462

[18] WHO. 2000 Severe *falciparum* Malaria. Trans. R. Soc. Trop. Med. 94:1–90.

[19] Naik, R. S. , O. H. Branch , A. S. Woods , M. Vijaykumar , D. J. Perkins , B. L. Nahlen , A. A. Lal , R. J. Cotter , C. E. Costello , C. F. Ockenhouse , et al. 2000 Glycosylphosphatidylinositol anchors of *Plasmodium falciparum*: molecular characterization and naturally elicited antibody response that may provide immunity to malaria pathogenesis. J. Exp. Med. 192:1563–1576. 11104799 10.1084/jem.192.11.1563 PMC2193092

[20] Naik, R. S. , G. Krishnegowda , C. F. Ockenhouse , and D. C. Gowda . 2006 Naturally elicited antibodies to glycosylphosphatidylinositols (GPIs) of *Plasmodium falciparum* require intact GPI structures for binding and are directed primarily against the conserved glycan moiety. Infect. Immun. 74:1412–1415. 16428795 10.1128/IAI.74.2.1412-1415.2006 PMC1360366

[21] Aribot, G. , C. Rogier , J. L. Sarthou , J. F. Trape , A. T. Balde , P. Druilhe , and C. Roussilhon . 1996 Pattern of immunoglobulin isotype response to *Plasmodium falciparum* blood‐stage antigens in individuals living in a holoendemic area of Senegal (Dielmo, west Africa). Am. J. Trop. Med. Hyg. 54:449–457. 8644897 10.4269/ajtmh.1996.54.449

[22] Trape, J. F. , C. Rogier , L. Konate , N. Diagne , H. Bouganali , B. Canque , F. Legros , A. Badji , G. Ndiaye , P. Ndiaye , et al. 1994 The Dielmo project: a longitudinal study of natural malaria infection and the mechanisms of protective immunity in a community living in a holoendemic area of Senegal. Am. J. Trop. Med. Hyg. 51:123–137. 8074247 10.4269/ajtmh.1994.51.123

[23] Perraut, R. , C. Joos , C. Sokhna , H. E. Polson , J. F. Trape , A. Tall , L. Marrama , O. Mercereau‐Puijalon , V. Richard , and S. Longacre . 2014 Association of antibody responses to the conserved *Plasmodium falciparum* merozoite surface protein 5 with protection against clinical malaria. PLoS ONE 9:e101737. 25047634 10.1371/journal.pone.0101737 PMC4105459

[24] Cissoko, Y. , M. Daou , K. E. Lyke , A. Dicko , I. Diarra , A. Kone , A. Guindo , K. Traore , G. Krishnegowda , D. A. Diallo , et al. 2006 Serum antibody levels to glycosylphosphatidylinositols in specimens derived from matched Malian children with severe or uncomplicated *Plasmodium falciparum* malaria and healthy controls. Am. J. Trop. Med. Hyg. 75:199–204. 16896119 PMC2738947

[25] de Souza, J. B. , J. Todd , G. Krishegowda , D. C. Gowda , D. Kwiatkowski , and E. M. Riley . 2002 Prevalence and boosting of antibodies to *Plasmodium falciparum* glycosylphosphatidylinositols and evaluation of their association with protection from mild and severe clinical malaria. Infect. Immun. 70:5045–5051. 12183551 10.1128/IAI.70.9.5045-5051.2002 PMC128284

[26] Mbengue, B. , B. Niang , B. Diatta , A. Tall , O. Garraud , R. Perraut , and A. Dieye . 2010 The use of crude *Plasmodium falciparum* antigens for comparison of antibody responses in patients with mild malaria vs. cerebral malaria. Iran J. Immunol. 7:150–161. 20876986 10.22034/iji.2010.17052

[27] Mbengue, B. , M. Sylla Niang , R. Ndiaye Diallo , G. Diop , A. Thiam , O. Ka , A. Toure , A. Tall , R. Perraut , and A. Dieye . 2015 IgG responses to candidate malaria vaccine antigens in the urban area of Dakar (Senegal): evolution according to age and parasitemia in patients with mild symptoms. Bull. Soc. Pathol. Exot. 108:94–101. 25925805 10.1007/s13149-015-0419-4

[28] Brasseur, P. , J. J. Ballet , and P. Druilhe . 1990 Impairment of *Plasmodium falciparum*‐specific antibody response in severe malaria. J. Clin. Microbiol. 28:265–268. 2179259 10.1128/jcm.28.2.265-268.1990 PMC269588

[29] Hunt, N. H. , and G. E. Grau . 2003 Cytokines: accelerators and brakes in the pathogenesis of cerebral malaria. Trends Immunol. 24:491–499. 12967673 10.1016/s1471-4906(03)00229-1

[30] Mbengue, B. , B. Diatta , B. Niang , N. Diagne , M. Ndiaye , L. Marrama , R. Perraut , and A. Dieye . 2011 Differential Kinetics of plasma procalcitonin levels in cerebral malaria in urban Senegalese patients according to disease outcome. Microbiol. Res. 2:80–84.

[31] Grau, G. E. , P. F. Piguet , P. Vassalli , and P. H. Lambert . 1989 Tumor‐necrosis factor and other cytokines in cerebral malaria: experimental and clinical data. Immunol. Rev. 112:49–70. 2575074 10.1111/j.1600-065x.1989.tb00552.x

[32] Molyneux, M. E. , T. E. Taylor , J. J. Wirima , and G. E. Grau . 1991 Tumour necrosis factor, interleukin‐6, and malaria. Lancet 337:1098. 1673519 10.1016/0140-6736(91)91745-g

[33] Sarthou, J. L. , G. Angel , G. Aribot , C. Rogier , A. Dieye , A. Toure Balde , B. Diatta , P. Seignot , and C. Roussilhon . 1997 Prognostic value of anti‐*Plasmodium falciparum*‐specific immunoglobulin G3, cytokines, and their soluble receptors in West African patients with severe malaria. Infect. Immun. 65:3271–3276. 9234786 10.1128/iai.65.8.3271-3276.1997 PMC175463

[34] de Souza, J. B. , M. Runglall , P. H. Corran , L. C. Okell , S. Kumar , D. C. Gowda , K. N. Couper , and E. M. Riley . 2010 Neutralization of malaria glycosylphosphatidylinositol in vitro by serum IgG from malaria‐exposed individuals. Infect. Immun. 78:3920–3929. 20566691 10.1128/IAI.00359-10 PMC2937451

[35] Hack, C. E , L. A Aarden , and L. G. Thijs . 1997 Role of cytokines in sepsis. Adv. Immunol. 66:101–195. 9328641 10.1016/s0065-2776(08)60597-0

[36] Coban, C. , K. J. Ishii , T. Kawai , H. Hemmi , S. Sato , S. Uematsu , M. Yamamoto , O. Takeuchi , S. Itagaki , N. Kumar , et al. 2005 Toll‐like receptor 9 mediates innate immune activation by the malaria pigment hemozoin. J. Exp. Med. 201:19–25. 15630134 10.1084/jem.20041836 PMC2212757

[37] Gowda, D. C. 2007 TLR‐mediated cell signaling by malaria GPIs. Trends Parasitol. 23:596–604. 17980663 10.1016/j.pt.2007.09.003

[38] McCall, M. B. , M. G. Netea , C. C. Hermsen , T. Jansen , L. Jacobs , D. Golenbock , A. J. van der Ven , and R. W. Sauerwein . 2007 *Plasmodium falciparum* infection causes proinflammatory priming of human TLR responses. J. Immunol. 179:162–171. 17579034 10.4049/jimmunol.179.1.162

[39] Gowda, N. M. , X. Wu , and D. C. Gowda . 2012 TLR9 and MyD88 are crucial for the development of protective immunity to malaria. J. Immunol. 188:5073–5085. 22516959 10.4049/jimmunol.1102143 PMC3345097

[40] Coban, C. , Y. Igari , M. Yagi , T. Reimer , S. Koyama , T. Aoshi , K. Ohata , T. Tsukui , F. Takeshita , K. Sakurai , et al. 2010 Immunogenicity of whole‐parasite vaccines against *Plasmodium falciparum* involves malarial hemozoin and host TLR9. Cell Host Microbe 7:50–61. 20114028 10.1016/j.chom.2009.12.003

[41] Couper, K. N. , T. Barnes , J. C. Hafalla , V. Combes , B. Ryffel , T. Secher , G. E. Grau , E. M. Riley , and J. B. de Souza . 2010 Parasite‐derived plasma microparticles contribute significantly to malaria infection‐induced inflammation through potent macrophage stimulation. PLoS Pathog. 6:e1000744. 20126448 10.1371/journal.ppat.1000744 PMC2813278

[42] Gowda, N. M. , X. Wu , S. Kumar , M. Febbraio , and D. C. Gowda . 2013 CD36 contributes to malaria parasite‐induced pro‐inflammatory cytokine production and NK and T cell activation by dendritic cells. PLoS ONE 8:e77604. 24204889 10.1371/journal.pone.0077604 PMC3810381

[43] Newton, P. N. , K. Chotivanich , W. Chierakul , R. Ruangveerayuth , P. Teerapong , K. Silamut , S. Looareesuwan , and N. J. White . 2001 A comparison of the in vivo kinetics of *Plasmodium falciparum* ring‐infected erythrocyte surface antigen‐positive and ‐negative erythrocytes. Blood 98:450–457. 11435316 10.1182/blood.v98.2.450

[44] Wu, X. , N. M. Gowda , S. Kumar , and D. C. Gowda . 2010 Protein‐DNA complex is the exclusive malaria parasite component that activates dendritic cells and triggers innate immune responses. J. Immunol. 184:4338–4348. 20231693 10.4049/jimmunol.0903824 PMC2851449

[45] Orengo, J. M. , A. Leliwa‐Sytek , J. E. Evans , B. Evans , D. van de Hoef , M. Nyako , K. Day , and A. Rodriguez . 2009 Uric acid is a mediator of the *Plasmodium falciparum*‐induced inflammatory response. PLoS ONE 4:e5194. 19381275 10.1371/journal.pone.0005194 PMC2667251

